# Delta- and gamma-tocotrienol isomers are potent in inhibiting inflammation and endothelial activation in stimulated human endothelial cells

**DOI:** 10.3402/fnr.v60.31526

**Published:** 2016-07-06

**Authors:** Suhaila Muid, Gabriele R. Anisah Froemming, Thuhairah Rahman, A. Manaf Ali, Hapizah M. Nawawi

**Affiliations:** 1 Faculty of Medicine, Universiti Teknologi MARA (UiTM), Selangor, Malaysia; 2 Institute of Pathology, Laboratory and Forensic Medicine, Universiti Teknologi MARA, Selangor, Malaysia; 3 Faculty of Agriculture & Biotechnology, Universiti Sultan Zainal Abidin, Kuala Terengganu, Malaysia

**Keywords:** tocotrienols, inflammation, endothelial activation, NF*κ*B, endothelial cells

## Abstract

**Background:**

Tocotrienols (TCTs) are more potent antioxidants than α-tocopherol (TOC). However, the effectiveness and mechanism of the action of TCT isomers as anti-atherosclerotic agents in stimulated human endothelial cells under inflammatory conditions are not well established.

**Aims:**

1) To compare the effects of different TCT isomers on inflammation, endothelial activation, and endothelial nitric oxide synthase (eNOS). 2) To identify the two most potent TCT isomers in stimulated human endothelial cells. 3) To investigate the effects of TCT isomers on NFκB activation, and protein and gene expression levels in stimulated human endothelial cells.

**Methods:**

Human umbilical vein endothelial cells were incubated with various concentrations of TCT isomers or α-TOC (0.3–10 µM), together with lipopolysaccharides for 16 h. Supernatant cells were collected and measured for protein and gene expression of cytokines (interleukin-6, or IL-6; tumor necrosis factor-alpha, or TNF-α), adhesion molecules (intercellular cell adhesion molecule-1, or ICAM-1; vascular cell adhesion molecule-1, or VCAM-1; and e-selectin), eNOS, and NFκB.

**Results:**

δ-TCT is the most potent TCT isomer in the inhibition of IL-6, ICAM-1, VCAM-1, and NFκB, and it is the second potent in inhibiting e-selectin and eNOS. γ-TCT isomer is the most potent isomer in inhibiting e-selectin and eNOS, and it is the second most potent in inhibiting is IL-6, VCAM-1, and NFκB. For ICAM-1 protein expression, the most potent is δ-TCT followed by α-TCT. α- and β-TCT inhibit IL-6 at the highest concentration (10 µM) but enhance IL-6 at lower concentrations. γ-TCT markedly increases eNOS expression by 8–11-fold at higher concentrations (5–10 µM) but exhibits neutral effects at lower concentrations.

**Conclusion:**

δ- and γ-TCT are the two most potent TCT isomers in terms of the inhibition of inflammation and endothelial activation whilst enhancing eNOS, possibly mediated via the NFκB pathway. Hence, there is a great potential for TCT isomers as anti-atherosclerotic agents.

Atherosclerosis is a slowly progressing disease of the medium- and large-sized arteries, characterized by the formation of fatty and fibrous lesions in the vessel wall (1). As a result, it will lead to serious atherosclerosis-related clinical complications such as stroke, peripheral vascular diseases (PVD), and coronary artery disease (CAD) (2). To date, CAD remains the major cause of mortality in the world, typically claiming one-third of all deaths (3). Inflammation and endothelial activation are the early stages in the development of atherosclerosis. During these stages, there will be an overexpression of pro-inflammatory cytokines (interleukin-6, or IL-6) and adhesion molecules (intercellular cell adhesion molecule-1, or ICAM-1; vascular cell adhesion molecule-1, or VCAM-1; and e-selectin) by the endothelial cells that is mediated via NFκB activation (4). Subsequently, there will be an increment of monocytes adhesion to endothelial cells. Therefore, it is suggested that IL-6, ICAM-1, VCAM-1, and e-selectin can be used as useful predictive biomarkers for atherosclerotic progression and new targets of treatment (5). Supplementation with anti-atherosclerotic agents with anti-inflammatory and anti-endothelial activation properties has the potential to prevent atherosclerosis-related complications. This is particularly so in human subjects with high risks, such as metabolic syndrome and hypercholesterolaemia (6).

Endothelial nitric oxide synthase (eNOS) generally has protective effects within the cardiovascular system (7). It has been indicated that eNOS plays a protective role in cerebral ischemia by preserving cerebral blood flow in eNOS knockout mice (8). The dramatic decrease of eNOS can occur during early stages of atherosclerosis leading to NO reduction (9). Nitric oxide (NO) modulates leukocyte-endothelial cell activation through the direct effect of NO on the regulation of cytokines and adhesion molecule expression by the transcription factor NFκB (10). NO induces the transcription of IκBα, an inhibitor of NFκB, thus stabilizing the inhibitory NFκB/IκBα complex in the cytosol (11). Another cellular protective mechanism of NO from oxidative stress is via its rapid interaction with superoxide, a pro-adhesive molecule (12).

Epidemiological studies have indicated the beneficial effects of vitamin E in the reduction of cardiovascular events, but in various clinical trials, the results were contradictory (13). On the other hand, tocotrienols (TCTs) have been postulated to be more cardioprotective than alpha-tocopherol (α-TOC) by virtue of their cholesterol-lowering property and better anti-oxidant activities (14–16). Human studies have reported the ability of TCTs to reverse the blockage of carotid artery and platelet aggregation and hence reduce the risk of atherosclerosis, stroke, and ischemic heart disease (17). Therefore, the above evidence makes TCTs an interesting candidate in vitamin E research. However, α-TOC has often been synonymously and incorrectly referred to as vitamin E, when vitamin E actually consists of both TOCs and TCTs. The existence of TCTs has almost always been disregarded in vitamin E research (18).

TOCs and TCTs are collectively called tocols (19). They are composed of a chromanol ring with an attached phytyl side chain. Both tocols consist of four isomers (α-, β, γ-, and δ-) according to the presence of methyl groups and all of them are active vitamers of vitamin E (20). TCTs differ from TOCs by possessing three double bonds in the phytyl side chain (21). High concentrations of TCTs are present in crude palm oil and extracts from the fruits of *Elaeis guineensis* and annatto plants (22, 23). The advantage of TCTs when compared with TOCs is that they are more potent anti-oxidant, anti-cancer, anti-aging, anti-thrombotic, and anti-angiogenic activities (24). However, data are still lacking on the effects of TCT isomers in the absence of TOCs (pure TCT) on inflammation and endothelial activation, particularly in endothelial cells (EC). Furthermore, the possible underlying mechanisms of the anti-inflammatory and anti-endothelial activation effects of TCTs are not well established.

Most TCT studies investigate the effects of TCT-TOC mixed fraction (TTMF), rather than the TCTs in the absence of TOCs, on inflammation in monocytes and macrophages. Furthermore, there are very few studies on the effects of TCT isomers on endothelial cell activation (25, 26). The few existing TCT studies on endothelial cells mainly focused on its benefits as an anti-angiogenic agent to halt tumor growth and new vascularization (24).

Although the activity of TCTs is superior to that of TOCs, the potential role of TCTs in the prevention of atherosclerosis has received minimal public attention. Furthermore, the data on TCTs and its potential against the development of atherosclerosis is still scarce. It has been suggested that TCTs are expected to accomplish as an important prevention option in atherosclerosis-related complications, such as CAD (27). In addition, determining the most effective TCT isomers is crucial to ensure effective scientific and clinical outcomes. Previously, we have reported the beneficial effects of TTMF in the reduction of inflammation and human endothelial cell activation (28). Therefore, in this present study, the effects of palm-oil-extracted different TCT isomers (α-, β-, γ-, δ-, and TCT) on inflammation and endothelial activation were investigated. The two most potent and effective TCT isomers as potential anti-atherosclerotics agents were identified. The effects of TCT isomers of NFκB activation were examined to determine whether anti-inflammatory and anti-endothelial activation is mediated via that NFkB pathway. This study also explored the effects of TCT isomers on eNOS in human endothelial cells.

## Materials and method

### Materials

Isomers of α-, β-, γ-, and δ-TCT (>97%) were provided by Davos Life Sciences, Singapore. Medium 200 and low-serum growth supplements (LSGS) were obtained from Cascade Biologics, Portland, Oregon, USA. RPMI-1640 medium (with glutamax-I and HEPES), L-glutamine, and fetal bovine serum (FBS) were purchased from Gibco-Life Technologies, Carlsbad, California, USA. Penicillin/streptomycin was purchased from PAA laboratories GmbH, Pasching, Austria. 3-(4,5-Dimethylthiazol-2-yl)-2,5-diphenyltetrazolium bromide (MTT) and dimethyl sulfoxide (DMSO) were purchased from Fluka, Darmstadt, Germany. Accutase was purchased from ICN Biomedical, Morgan Irvine, California, USA. Phosphate buffer saline (PBS) was obtained from MP Biomedicals, Strasbourg, France. ELISA test kits for IL6, tumor necrosis factor-alpha (TNF-α), sICAM-1, sVCAM-1, and e-selectin were purchased from Bender Medsystems, Vienna, Austria. The NFκB binding assay kit was obtained from Cayman Chemicals, Ann Arbor, Michigan, USA. The Quantikine eNOS immunoassay kit was manufactured by R&D BioSystems, Minneapolis, Minnesota, USA. The tgRNA extraction kit and Sensiscript Reverse Transcription kit was manufactured by Qiagen, Valencia, California, USA. Agilent RNA 6,000 Pico was manufactured by Agilent Technologies, Waldbronn, Germany. Primers for quantitative real-time polymerase chain reaction (qPCR) assay were produced by First BASE Laboratories, Seri Kembangan, Selangor, Malaysia. SYBR Green for qPCR assay was obtained from Bio-Rad Laboratories, Hercules, California. Rose Bengal staining was purchased from Sigma Aldrich, St. Louis, Missouri, USA. All chemicals used in this assay were tissue-culture grade.

### Cell culture

Human umbilical vein endothelial cells (HUVECs) were purchased from Cascade Biologics, USA. HUVECs at passage 4–7 were cultured in medium 200, supplemented with LSGS in a humidified incubator set at 37°C and 5% carbon dioxide (CO_2_) until confluent. Cells were grown in 25-cm^2^ culture flasks (BD Falcon, UK). Cells were harvested by cell detachment solution (Accutase) and sub-cultivation ratio was 1:3 (culture: medium). All chemicals used in this assay were tissue-culture grade.

### Preparation of different concentrations of TCT isomers and α-TOC in cell culture medium

A stock solution of α-, β-, γ-, and δ-TCT isomers or α-TOC was prepared in absolute ethanol and stored at −80°C for not more than 3 days. The sample stock solutions were then mixed with FBS at a ratio of 1:20 and incubated at 37°C for 15 min, during which time a brief vortex was conducted every 5 min. Following this, a working solution of each sample was prepared in RPMI-1640 culture medium. These working solutions were further diluted with RPMI-1640 to obtain the desired concentrations needed in each assay (0.3–10 uM), based on our previous experiment (28). The final ethanol concentration in each isomer of either α-, β-, γ-, or δ-TCT isomers and controls (unstimulated and lipopolysaccharides (LPS) alone) in the assay plate was 0.004%. The final FBS concentrations in all samples were standardized to 8%.

### Enzyme-link immunosorbent assay

Concentrations of soluble protein levels of inflammation markers in a supernatant of HUVEC cells were measured by enzyme-link immunosorbent assay (ELISA) standard kit (Bender Medsystem). Tests were performed according to the instructions provided by the manufacturer. At the end of ELISA testing, absorbance was obtained by spectrophotometer (Micro Quant, Biotek Instruments, Winooski, Vermont, USA) at 405-nm wavelength.

### Quantitation of NFκB (p50) protein levels in cell lysates

After incubation of α-, β-, γ-, or δ-TCT isomers, together with LPS (1 µg/mL), for 16 h, HUVECs were collected to perform nuclear extraction (Cayman Chemicals). The quantitation of NFκB (p50) protein binding in nuclear extract was performed by the ELISA method according to the manufacturer's instruction (Cayman Chemicals). The absorbance in each well was measured at 450 nm with a microplate reader (Tecan Safire, Männedorf, Switzerland).

### Quantitation of eNOS protein level in cell lysates

Quantitation of eNOS protein level in cell lysates was performed with a commercially available eNOS immunoassay kit (R&D BioSystems, USA). The test was performed according to manufacturer's protocol. The optical density was determined using a microplate reader (Tecan Safire, Männedorf, Switzerland) set at 450 nm and reference wavelength at 570 nm.

### IL-6, TNF-α, ICAM-1, VCAM-1, e-selectin, NFκB, and eNOS gene expression by quantitative real time PCR

Total RNA was then extracted from the cell pellets using the RNA extraction kit from Qiagen, USA. RNA purity and concentration was determined by an automated capillary electrophoresis system, Agilent 2100 Bioanalyzer (Agilent Technologies, Santa Clara, California, USA). Total RNA extract samples were reverse transcribed to cDNA using the Sensiscript kit (Qiagen, USA). After that, 1 µL of cDNA of every sample was added into 24 µL of reaction mixture containing primer pairs of forward and reverse SYBR green fluorescence dye and RNAse free water. PCR was then performed in MyIQ thermal cycler (Bio-Rad Laboratories, Hercules, USA), starting with the denaturing process at 95°C for 10 s. The annealing process was carried out with the temperature and duration according to Table 2 and continued with the extension process at 72°C for 30 s. These whole processes were repeated for 49 cycles. The melt curve was obtained for each run to ensure the purity of primer. A standard curve graph was produced for each run to obtain PCR efficiency. Relative gene expression (fold-change) was calculated using the 2^−ΔΔCT^ method with the Genex software (Bio Rad Laboratories, Hercules, California, USA). GAPDH was selected as a reference gene. The primer sequence, annealing temperature, and duration for each measured gene are presented in Table 1.

**Table 1:** Primer sequence, annealing temperature (T°), and annealing time for each measured gene using quantitative real time PCR (qPCR)

Parameters	Primer sequence	Annealing T° (°C)	Annealing time (s)
IL-6 (358 bp)	Forward primer: GCC TTC GGT CCA GTT GCC TT	60	24
	Reverse primer: GCA GAA TGA GAT GAG TTG TC	60	24
ICAM-1 (406 bp)	Forward Primer: AGAGGTCTCAGAAGFGGACCG	58	45
	Reverse Primer: GGGCCATACAGGACACGAAG	58	45
VCAM-1 (460 bp)	Forward primer: GGTGGGACACAAATAAGGGTTTTGG	60	45
	Reverse primer: CTTGCAATTCTTTTACAGCCTGCC	60	45
E-selectin (244 bp)	Forward primer: TGAAGCTCCCACTGAGTCCAA	60	45
	Reverse primer: GGTGCTAATGTCAGGAGGGAGA	60	45
TNF-α (84 bp)	Forward primer: CCGGGCGTGGTGGTGAG	60	45
	Reverse primer: TCTGCCTTTTGGGTCTTGTGAATA	60	45
NFκB (p50) (376 bp)	Forward primer: AAC CTG CAG ACT CCA CT	65	45
	Reverse primer: ACA CCA GGT CAG GAT TTT GC	65	45
eNOS (418 bp)	Forward primer: ATGGGCAACTTGAAGAGCGTGG	55.8	45
	Reverse primer: TAGTACTGGTTGATGAAGTCCC	55.8	45
HPRT-1 (523 bp)	Forward primer: GGCAAAACAATGCAAACCTT	60	45
	Reverse primer: CAAGGGCATATCCTACGACAA	60	45
GAPDH (472 bp)	Forward primer: CCACCCATGGCAAATTCCATGGCA	60	45
	Reverse primer: TCTAGACGGCAGGTCAGGTCCACC	60	45

### Statistical analysis

Results are expressed as mean±SD. The analysis of variance (ANOVA) with *post hoc* test was performed. The differences between each concentration of TCT isomers and LPS controls were analysed with the Bonferroni *post hoc* analysis. All data were analyzed by a statistical package program, SPSS version 16.0. The level of significance was set at *p*<0.05. To compare the effectiveness and identify the two most potent TCT isomers, the area under the curve (AUC) analysis for each TCT isomers, across all concentrations combined (0.3–10 µM), was performed for each biomarker using the Graph Version 4.3 software. The AUC analysis was used because the effects of TCT isomers on each marker of inflammation and endothelial activation were not in the dose-dependent manner. After obtaining the AUC for each TCT isomer, the percentage (%) inhibition against their respective controls was calculated for each biomarker.

## Results

### Effects of TCT isomers on IL-6 protein levels and gene expression

**Fig. 1:**
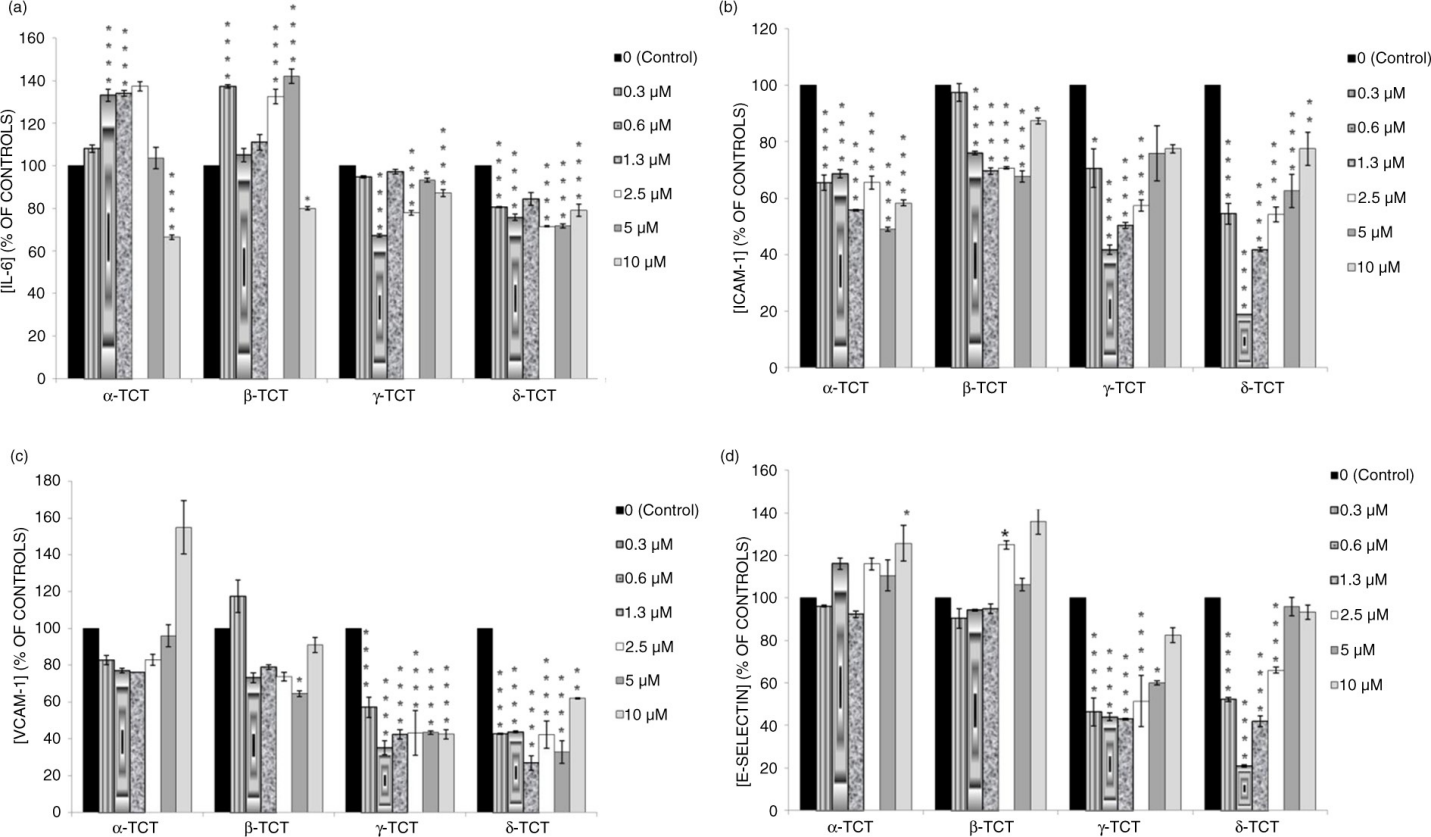
Effects of TCT isomers (0.3–10 µM) on the (a) IL-6, (b) ICAM-1, (c) VCAM-1, and (d) e-selectin protein expression in LPS-stimulated HUVECs. Prior to incubation, e-selectin protein expression in the supernatant was measured by ELISA. Results are expressed as a percentage (%) of LPS controls. Data are expressed as mean±SD (*n*=3). *p<0.05, **p<0.01 and ****p<0.0001 compared to HUVECs incubated with LPS alone.

The effects of TCT isomers on the secretion of IL-6 in LPS-stimulated HUVECs are illustrated in Fig. 1a. α- and β-TCT at 10 µM concentration had significantly lower IL-6 levels compared to LPS stimulated HUVECs. However, α- and β-TCT at lower concentrations had enhanced IL-6 levels significantly compared to controls. γ- and δ-TCT across most concentrations (0.3–10 µM) significantly reduced IL-6 levels compared to LPS controls. AUC analysis showed that the two most potent TCT isomers for IL-6 inhibition were δ-TCT (25.6%) followed by γ-TCT (12.0%) (Table 2).

**Table 2:** Percent of inhibition of inflammation and endothelial activation biomarkers, NFκB and eNOS by TCT isomers and based on area under the curve (AUC) analysis

	IL-6 % INHIBIT		TNF-α % INHIBIT		ICAM-1 % INHIBIT		VCAM-1 % INHIBIT		E-SEL % INHIBIT		NFκB % INHIBIT		eNOS % INCREMENT	
							
	P	G	P	G	P	G	P	G	P	G	P	G	P	G
α-TCT	−8.2	**36.1**	−7.8	−8.2	**37.0**	**50.5**	−7.8	43.3	−15.4	44.3	17.3	28.4	19.2	169.1
β-TCT	−22.5	24.7	8.7	−114.4	28.2	48.5	27.8	**45.4**	−17.9	**64.9**	16.0	**37.7**	8.3	135.1
γ-TCT	**12.0**	−33.0	−7.4	−74.2	31.7	34.0	**57.6**	−76.3	**38.4**	45.4	**22.2**	−10.3	**137.5**	**857.7**
δ-TCT	**25.6**	**73.2**	**6.9**	−**1.0**	**38.8**	**76.3**	**59.3**	**88.7**	**17.7**	**60.8**	**22.4**	**53.6**	**111.2**	**396.9**

INHIBIT, inhibition; P, protein; G, gene; TCT, tocotrienol; bold, first and second most potent TCT isomers.

### Effects of TCT isomers on TNF-α protein secretion

The effects of TCT isomers on TNF-α protein secretion were investigated in LPS-stimulated HUVECs (data not shown). TNF-α protein secretion was unaffected by the coincubation of TCT isomers with LPS across all concentrations (0.3–10 µM).

### Effects of TCT isomers on ICAM-1 protein secretion

The effects of TCT isomers on ICAM-1 protein secretion in LPS-stimulated HUVECs are illustrated in Fig. 1b. In comparison to LPS alone, the coincubation of LPS with α-, β-, γ-, and δ-TCT at concentrations of 0.3–10 µM significantly inhibits ICAM-1 levels compared to controls. AUC analysis showed that the two most potent TCT isomers for ICAM-1 inhibition were δ-TCT (38.8%), followed by α-TCT (37.0%) (Table 2).

### Effects of TCT isomers on VCAM-1 protein secretion

Figure 1c illustrates the effects of TCT isomers on VCAM-1 protein secretion in LPS- stimulated HUVECs. In-comparison to LPS alone, the co-incubation of LPS and γ- and δ-TCT at concentrations of 0.3–10 µM significantly inhibits the VCAM-1 levels in HUVECs (35.1±3.7% – 57.1±5.4% versus 100.0±0.0%, *p*<0.0001). In contrast, there was no significant VCAM-1 reduction by either α-TCT or β-TCT isomer. AUC analysis showed that the two most potent TCT isomers for VCAM-1 protein expression inhibition were δ-TCT (59.3%), followed by γ-TCT (57.6%) (Table 2).

### Effects of TCT isomers on e-selectin protein secretion

Figure 1d shows the effects of TCT isomers on e-selectin levels in LPS-stimulated HUVECs. The production of e-selectin was significantly reduced by the coincubation of LPS, together with γ- and δ-TCT, at most concentrations (0.3–2.5 µM), compared with the results from LPS alone. There was no significant reduction in e-selectin production in LPS-stimulated HUVECs compared to controls with α- and β-TCT. It is noted that, at high concentrations of 10 µM, both α- and β-TCT led to significantly higher e-selectin production compared to controls. AUC analysis showed that the two most potent TCT isomers for e-selectin protein expression inhibition were γ-TCT (38.4%), followed by δ-TCT (17.7%) (Table 2).

### Effects of TCT isomers on eNOS protein expression in LPS-stimulated HUVEC

Figure 3a shows the effects of TCT isomers on eNOS expression in LPS-stimulated HUVECs. There was no beneficial effect in terms of the increment of eNOS protein expression by α- and β-TCT in LPS-stimulated controls. There was increment of eNOS production by γ-TCT at the higher concentrations of 5 and 10 µM compared to LPS controls. The coincubation of LPS and δ-TCT at lower concentrations (1.3 and 2.5 µM) significantly increased the production of eNOS in HUVECs. AUC analysis showed that the two most potent TCT isomers for increment in eNOS protein expression were γ-TCT (137.5%), followed by δ-TCT (111.2%) (Table 2).

### Effects of TCT isomers on NFκB activation in LPS stimulated HUVECs

Figure 3c shows the effects of TCT isomers on NFκB activation in LPS-stimulated HUVECs. Most concentrations of α-TCT, except for 0.6 µM, reduced the NFκB activation when compared to untreated LPS-stimulated controls. β-TCT across all concentrations (0.3–10 µM) showed no reduction in NFκB activation. There was a significant reduction in NFκB activation by γ- and δ-TCT across all concentrations (0.3–10 µM) compared to LPS-stimulated HUVECs. AUC analysis showed that the two most potent TCT isomers for NFκB deactivation were δ-TCT (22.4%) followed by γ-TCT (22.2%) (Table 2).

### Effects of TCT isomers on IL-6 gene expression in LPS-stimulated HUVECs

**Fig. 2:**
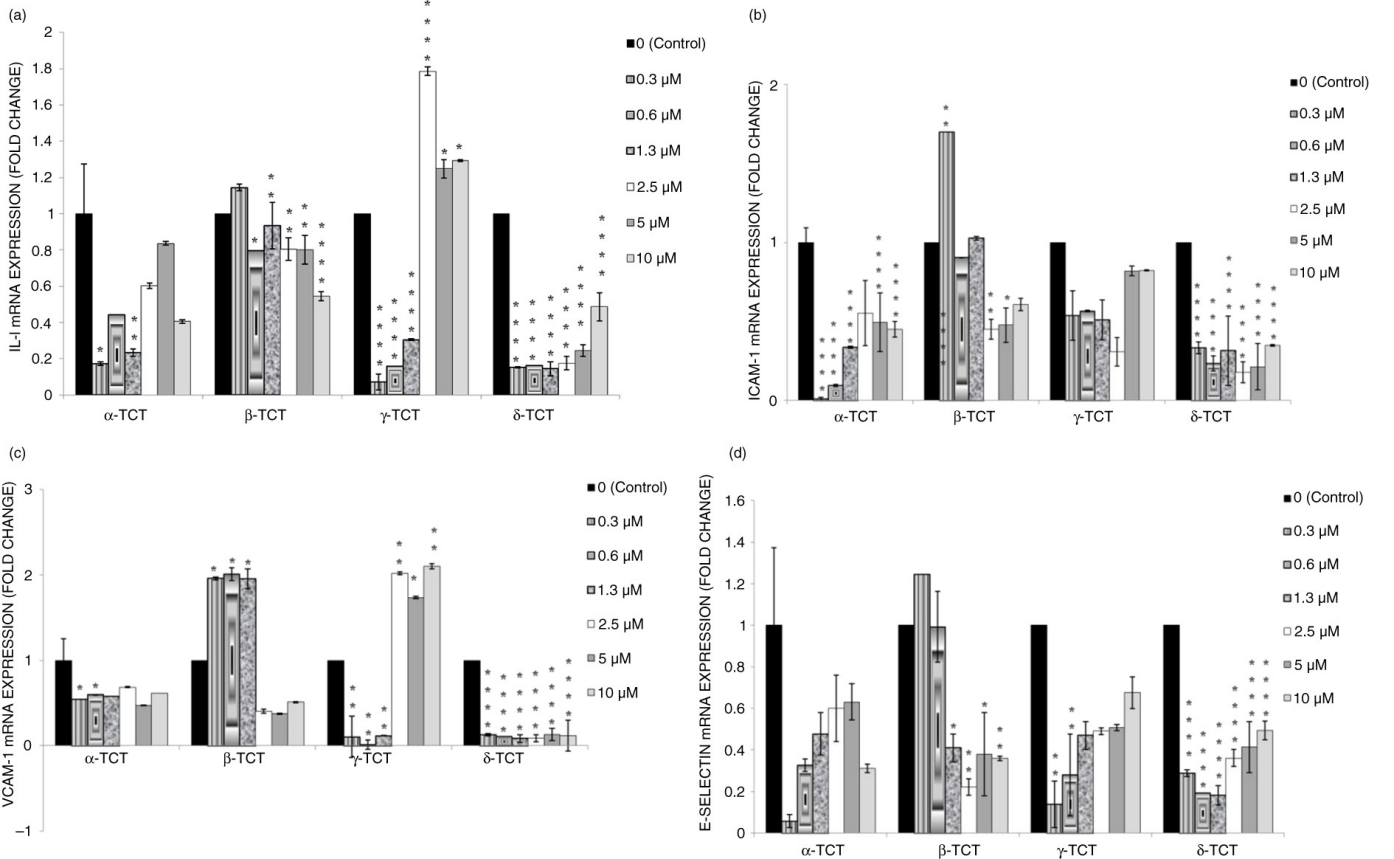
Effects of TCT isomers (0.3–10 µM) on the (a) IL-6, (b) ICAM-1, (c) VCAM-1, and (d) e-selectin gene expression in LPS-stimulated HUVECs. Prior to incubation, total RNA was extracted from the cells and subjected to quantitative real-time PCR (qPCR) to determine the ICAM-1 gene expression. Each data was normalized to 1.0 (HUVECs incubated with LPS alone) and GAPDH reference gene. Data are expressed as mean±SD. *p<0.05, **p<0.01 and ****p<0.0001 compared to HUVECs incubated with LPS alone.

Figure 2a illustrates the effects of TCT isomers on IL-6 gene expression in LPS-stimulated HUVECs. α-TCT at lower concentrations (0.3 and 1.3 µM) significantly down-regulated the IL-6 mRNA expression. The coincubation of LPS with β-TCT at almost all concentrations (0.6–10 µM) reduced the gene expression of IL-6. The coincubation of LPS, together with γ-TCT at lower concentration (0.3–1.3 µM), reduced the IL-6 mRNA expression. However, at higher concentrations (2.5–10 µM), γ-TCT lead to increased IL-6 gene expression. δ-TCT across all concentrations (0.3–10 µM) suppressed the IL-6 gene expression. AUC analysis showed that the two most potent TCT isomers for IL-6 gene expression down-regulation were δ-TCT (73.2%), followed by α-TCT (36.1%) (Table 2).

### Effects of TCT isomers on TNF-α gene expression in LPS-stimulated HUVECs

The coincubation of LPS with γ-TCT at low concentrations (0.3–1.3 µM) down-regulated the TNF-α gene expression compared to LPS controls. However, at higher concentrations (5–10 µM), γ-TCT appeared to enhance TNF-α gene expression. The coincubation with α-TCT, β-TCT, and δ-TCT, together with LPS, had no significant TNF-α reduction.

### Effects of TCT isomers on ICAM-1 gene expression in LPS-stimulated HUVECs

Figure 2b shows the effects of TCT isomers on ICAM-1 gene expression in LPS- stimulated HUVECs. α- and δ-TCT across all concentrations (0.3–10 µM) significantly suppressed ICAM-1 mRNA expression. The incubation of LPS with β-TCT at 2.5 and 5 µM concentrations reduced ICAM-1 gene expression compared the LPS controls. There was a reducing trend of ICAM-1 mRNA expression by the coincubation of γ-TCT with LPS but it did not reach a statistical significant level. AUC analysis showed that the two most potent TCT isomers for ICAM-1 gene expression down regulation were δ-TCT (76.3%), followed by α-TCT (50.5%) (Table 2).

### Effects of TCT isomers on VCAM-1 gene expression in LPS-stimulated HUVECs

Figure 2c shows the effects of TCT isomers on VCAM-1 mRNA expression in LPS- stimulated HUVECs. The coincubation of LPS and α-TCT down-regulated VCAM-1 mRNA expression compared to LPS alone at 0.3–0.6 µM. The coincubation of LPS with low concentrations of β-TCT (0.3–1.3 µM) increased the VCAM-1 mRNA expression compared to controls, whilst at higher concentration (2.5–10 µM) reduced VCAM-1 gene expression. In contrast, the coincubation of LPS, together with γ-TCT at lower concentration (0.3–1.3 µM), significantly suppressed the VCAM-1 mRNA expression compared to LPS alone, but at higher concentrations (2.5–10 µM), γ-TCT increased the VCAM-1 mRNA expression compared to HUVECs stimulated with LPS alone. δ-TCT across all concentrations (0.3–10.0 µM), coincubated with LPS, showed significant suppressive effects on the VCAM-1 mRNA expression compared to LPS controls. AUC analysis showed that the two most potent TCT isomers for VCAM-1 gene expression down regulation were δ-TCT (88.7%) followed by β-TCT (45.4%) (Table 2).

### Effects of TCT isomers on e-selectin gene expression in LPS-stimulated HUVECs

Figure 2d illustrates the effects of TCT isomers on e-selectin gene expression in LPS-stimulated HUVECs. There was no effect of α-TCT on e-selectin mRNA expression. β-TCT at 1.3–10 µM down-regulated e-selectin mRNA expression compared to LPS controls. The coincubation of LPS with γ-TCT at 0.3 and 0.6 µM concentrations reduced the e-selectin gene expression compared the LPS. δ-TCT across all concentrations (0.3–10 µM) suppressed e-selectin gene expression compared to LPS controls. AUC analysis showed that the two most potent TCT isomers for the down regulation of e-selectin gene expression were β-TCT (64.9%) followed by δ-TCT (60.8%) (Table 2).

### Effects of TCT isomers on eNOS gene expression in LPS-stimulated HUVECs

**Fig. 3:**
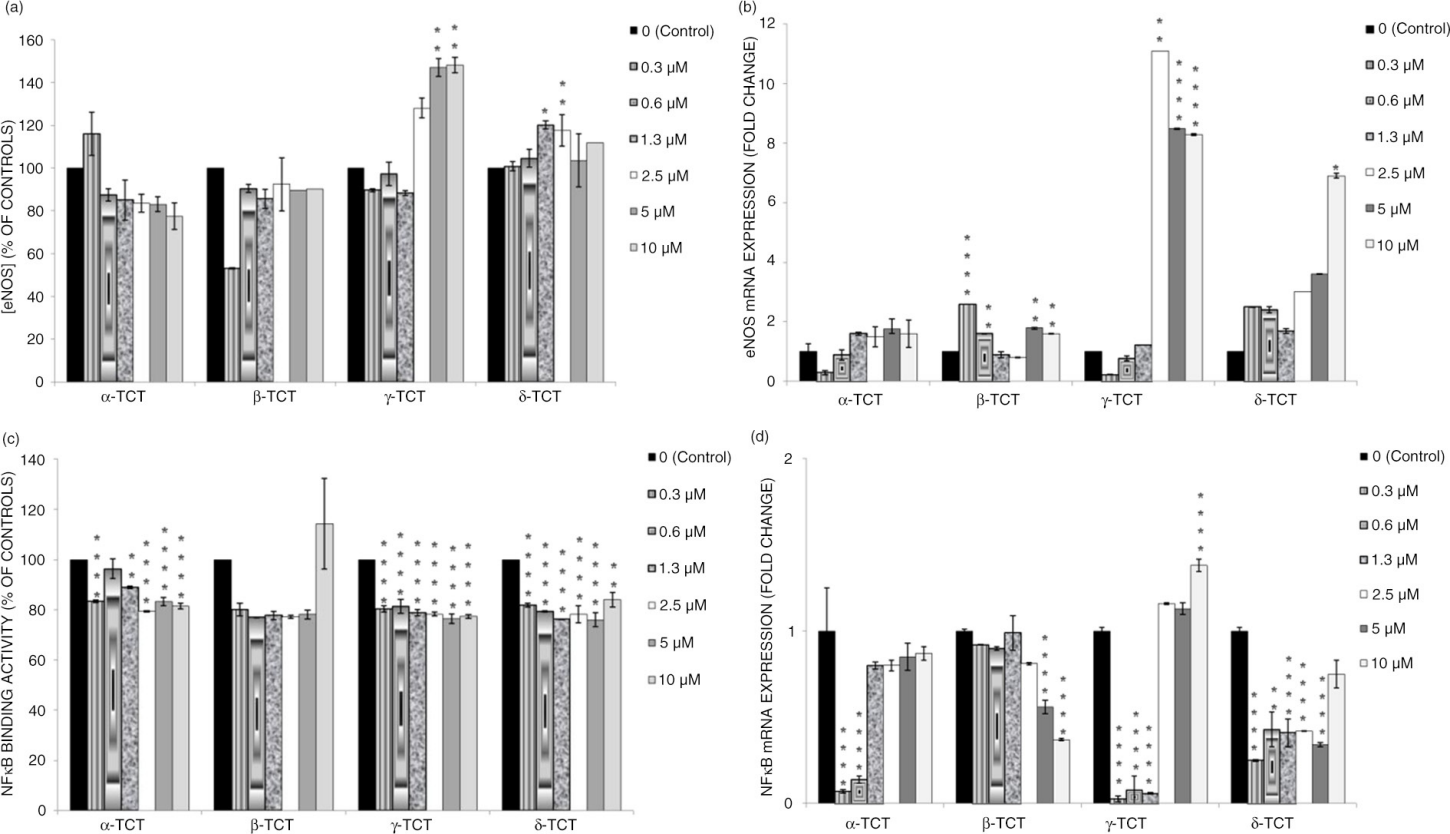
Effects of TCT isomers (0.3–10 µM) on the (a) eNOS protein expression, (b) eNOS gene expression, (c) NFκB protein expression, and (d) NFκB gene expression in LPS-stimulated HUVECs. Data are expressed as mean±SD (*n*=3). *p<0.05, **p<0.01 and ****p<0.0001 compared to HUVECs incubated with LPS alone.

Figure 3b shows the effects of TCT isomers on eNOS gene expression in LPS- stimulated HUVECs. α-TCT coincubated with LPS showed no effects on eNOS gene expression compared to controls. There was an increment in eNOS gene expression by β-TCT at the concentrations of 0.3–0.6 µM, compared to controls. The coincubation of LPS and γ-TCT at higher concentration (2.5–10 µM) showed a marked increment of eNOS gene expression compared to LPS alone. There was an upregulation of eNOS gene expression by the coincubation of δ-TCT and LPS at 10 µM, compared to LPS controls. AUC analysis showed that the two most potent TCT isomers for e-NOS gene expression down regulation were γ-TCT (857.7%), followed by δ-TCT (296.9%) (Table 2).

### Effects of TCT isomers on NFκB gene expression in LPS-stimulated HUVECs

Figure 3d shows the effects of TCT isomers on NFκB gene expression in LPS- stimulated HUVECs In comparison to LPS alone, the coincubation of LPS and α-TCT at lower concentrations (0.3–0.6 µM) blocked NFκB gene expression. The coincubation of LPS with β-TCT at higher concentrations (5–10 µM) lead to reduced NFκB gene expression compared to LPS alone, but not at lower concentrations (0.3–2.5 µM). The coincubation of LPS, together with γ-TCT at low concentrations (0.3–1.3 µM), blocked the NFκB expression compared to LPS alone. δ-TCT at 0.3–5.0 µM coincubated with LPS showed the suppressive effect on the NFκB gene expression compared to LPS controls. However, both α- and γ-TCT at higher concentrations did not lead to reduced NFκB gene expression. AUC analysis showed that the two most potent TCT isomers for NFκB gene expression down regulation are δ-TCT (53.6%), followed by β-TCT (37.7%) (Table 2).

## Discussion

Data on the effects of individual TCT isomers in the absence of TOC (Pure TCT) on inflammation and endothelial activation were scarce and not well established; hence, they were investigated in this present study. In addition, studies on the effects of TCT isomers in endothelial cells are limited, and several investigators have studied these effects on other cell types, including macrophages (26, 29). Given that endothelial activation leading to dysfunction is pivotal in atherogenesis, this present study has focused on their effects in human endothelial cells. Furthermore, although other investigators have reported the anti-inflammatory and anti-endothelial activation effects of TCT, the biomarkers reported have been limited, not covering most biomarkers of early atherogenesis, unlike this present study.

The main finding of this study is that, in general, all individual TCT isomers are beneficial in inhibiting IL-6, ICAM-1, VCAM-1, and e-selectin protein and gene expression in LPS-stimulated endothelial cells whilst enhancing eNOS. Amongst the four TCT isomers, δ-, followed by γ-TCT, are the two most potent isomers as anti-atherosclerotic agents. Previously, we have reported that TTMF (70% TCT: 30% α-TOC) at 0.3–10 µM reduced human endothelial IL-6, ICAM-1, VCAM-1, and e-selectin protein expression via the NFkB activation pathway, and increased eNOS (30).

Similar findings were reported by other studies in which TCT isomers reduced IL-6 and gene protein expression in LPS-stimulated macrophages and murine mammary cancer cells with δ-TCT being the most potent, followed by γ-TCT (24, 31). Previously, our group has also reported that TTMF at a lower concentration (0.3 µM) led to a reduction in IL-6 protein expression (28). With regards to ICAM-1 expression, δ-TCT is the most potent isomer followed by α-TCT. Theriault has shown that α-TCT inhibits ICAM-1 gene expression, but it was not compared with the other isomers. For VCAM-1 gene expression, δ-TCT is the most potent isomer followed by β-TCT. The latter agrees with the results reported by Naito et al. (26). For the inhibition of e-selectin protein expression, γ-TCT was shown to be the most potent TCT isomer, followed by δ-TCT. This is in contrast to that reported by Choa et al. (32), which showed that, in terms of e-selectin protein reduction, δ-TCT is the most potent isomer followed by γ-TCT in TNF-α stimulated HUVECs. Similarly, these discrepancies are possibly attributed to the different stimulants used.

However, for TNF- α, only γ-TCT at lower concentrations (0.3–2.5 µM) significantly reduced TNF-α gene expression in LPS-stimulated endothelial cells. This is in contrast to the findings of Qureshi et al. (2010), who indicated that some TCT isomers (α, γ, δ) inhibited TNF-α protein expression in LPS-stimulated macrophages. In addition, Yam et al. (31) reported that only α-TCT, but not the other isomers, showed a reduction in TNF-α protein expression in LPS-stimulated macrophages. This may be due to the different cell cultures used, where human endothelial cells were used in this present study, compared to macrophage in the previous studies.

However, at certain concentrations, cotreatment of α- and β-TCT with LPS has different effects in the production of ICAM-1, VCAM-1, and e-selectin. For ICAM-1, levels were reduced by α- and β-TCT across all concentrations (0.3–10 ug/mL). In contrast, α- and β-TCT showed a lack of beneficial effects in terms of the inhibition of VCAM-1 and e-selectin protein expression. This is discordant to the report of Theriault et al. that α-TCT reduced VCAM-1 and e-selectin protein expression in TNF-α stimulated endothelial cells. Similarly, Naito et al. showed that α-, β-, γ-, and δ-TCT isomers at 10 µM inhibited VCAM-1 expression in 25-hydroxycholesterol stimulated endothelial cells. In our experimental model, 10 µM γ- and δ-TCT inhibited VCAM-1 and e-selectin expression, whilst α- and β-TCT failed to do so. The discrepancy between the results of this present study and other work may possibly be explained by the different stimulants acting via different pathways. For example, LPS has been showed to act via NFκB, whilst TNF-α and 25-hydroxycholesterol act via the protein prenylation pathway (25, 26, 30). Other investigators have also suggested that the effects caused by a particular mediator depend on the nature of stimulators and target cell types (30).

This present study also shows that IL6, VCAM-1, and NFκB gene expression is upregulated by γ-TCT at the higher concentrations. The mechanisms is still unclear, however, it has been previously reported that gamma TCT is the most potent TCT isomers that beneficial as an anti-cancer agent (33). The results of this study suggest that γ-TCT at low concentrations—and not at high concentrations—are beneficial in the reduction of inflammation, VCAM-1, and NFκB activation in stimulated endothelial cells.

eNOS is expressed in vascular endothelial cells, especially at the endothelial layer of medium- to large-sized blood vessels (7). eNOS plays an important role in the NO production by ECs (34). NO modulates vascular tone, inhibits platelet function, prevents the adhesion of leukocytes, and reduces the proliferation of the intima (35). NO also functions as an anti-inflammatory agent and depresses the expression of adhesion molecules (12).

Das et al. (36) and Ikeda et al. (37) have reported that γ-TCT at 10 µM upregulates eNOS gene and protein expression. In addition, Das et al. (2005) showed that amongst all TCT isomers, γ-TCT was the most potent isomer. The findings of the present study are in the agreement with the above-mentioned previous studies. Both the present study and that by Das et al. examined all TCT isomers, but only one concentration (10 µM) was used by the latter whilst this study examined several concentrations. In contrast, Ikeda et al. only investigated one concentration of one isomer (γ-TCT). Furthermore, we found that δ-TCT, at a moderate concentration, also upregulates both eNOS protein and gene expression, which has not been previously reported. In summary, this study showed that γ-TCT was the most potent isomer in increasing eNOS protein and gene expression followed by delta TCT.

It is suggested that IL-6 can lead to a decrease of eNOS expression and contributes to the attenuation of NO production and the progression of atherosclerosis (38). Similarly, our finding reported the ability of TCT isomers in the reduction of IL-6 and at the same time increases eNOS.

The activation of NFkB leads to an increased expression of IL-6, ICAM-1, and VCAM-1. In this study, we found a reduced NFкB concentration in the nuclear cell lysates, implying reduced NFkB activation by TCTs. α- (in almost all concentrations), γ-, and δ-TCT isomers reduced NFκB protein expression in LPS-stimulated HUVECs. There was a down regulation of NFκB gene expression by γ- and δ-TCT in almost all concentrations. The AUC analysis indicated that δ-TCT is the most potent TCT isomer in NFκB protein expression reduction and was closely followed by γ-TCT. Previously, α-TCT was reported to effectively reduce NFκB activation in TNF-α stimulated endothelial cells (25). It has been suggested that TCT blocks the NFκB activation through the ability of TCT in inhibiting the phosphorylation of IκB by IKK complex, leading to a decrease of IκB complex degradation (39). When the IκB complex degradation is decreased, the translocation of NFκB into the nucleus is also being suppressed, leading to a further reduction of NFκB activation (40).

In comparison with the other TCT isomers, this study demonstrated that δ-TCT followed by γ-TCT are the most potent and effective anti-inflammatory and anti-endothelial activation agents. In this study, it has been shown that the effective anti-inflammatory and anti-endothelial activation action of δ-TCT and γ-TCT are mediated by the NFκB pathway. Other investigators have also demonstrated that the potency of δ-TCT and γ-TCT are due to the number of methyl group, which abolishes regulatory activity with respect to the HMG-CoA reductase degradation and sterol regulatory element-binding proteins (SREBP-2) processing (41). Naito et al. has demonstrated that the highest intracellular concentration of Vitamin E analogues in endothelial cells is δ-TCT compared to α-TCT and γ-TCT (26). These suggest that the superior anti-endothelial activation property of TCT isomers is possibly due to their intracellular concentrations (42).

## Conclusion

Overall, δ-TCT is the most potent TCT isomer followed by γ-TCT. δ-TCT has been clearly shown in this study to be the most potent isomer in inhibition of IL-6, ICAM-1, VCAM-1 expression, and NFκB and the second most potent in inhibiting e-selectin and enhancing eNOS expression. γ-TCT, on the other hand, is most potent isomer in term of inhibiting e-selectin expression and enhancing eNOS, whilst being the second most potent after δ-TCT in inhibiting IL-6 and VCAM-1 expression and NFκB. The ability of TCT isomers to reduce inflammation and endothelial activation is mediated via the NFκB pathway. TCT isomers may have great potential as an anti-atherosclerotics agent via their anti-inflammation and anti-endothelial activation property. This study adds knowledge to the scarcity of published studies on TCTs and their anti-inflammatory effects in relation to atherosclerosis. To date, studies on the anti-inflammatory effects of TCTs were more focused on cancer chemoprevention and treatment but very few have reported on TCTs as an anti-atherosclerotic agent. Therefore, it is timely for TCT isomers to be evaluated clinically to fully explore their potential in view of numerous *in vitro* and *in vivo* studies, and very limited human intervention clinical trials. Therefore, given the important roles of inflammation and endothelial activation in the pathogenesis of atherosclerosis, and by virtue of the potent anti-inflammatory and anti-endothelial activation effects of TCTs, there is a strong potential for TCTs in the prevention or regression of atherosclerosis and thus as an anti-atherosclerotic agent. TCT may have potential for those at risk of atherosclerosis or with atherosclerosis-related diseases such as CAD, PVD, stroke, and metabolic syndrome. Hence, future studies are warranted to investigate the use of TCTs in these conditions.
